# In Vitro Antioxidant, Photoprotective, and Volatile Compound Profile of Supercritical CO_2_ Extracts from Dandelion (*Taraxacum officinale* L.) Flowers

**DOI:** 10.3390/plants15010099

**Published:** 2025-12-28

**Authors:** Janina Sutkaitienė, Michail Syrpas, Petras Rimantas Venskutonis, Vaida Kitrytė-Syrpa

**Affiliations:** Department of Food Science and Technology, Kaunas University of Technology, Radvilėnų Rd. 19, LT-50254 Kaunas, Lithuaniamichail.syrpas@ktu.lt (M.S.); rimas.venskutonis@ktu.lt (P.R.V.)

**Keywords:** dandelion, supercritical CO_2_ extraction, flavour compounds, antioxidant activity, photoprotection

## Abstract

This study aimed to develop a sustainable approach for isolating bioactive lipophilic components from *Taraxacum officinale* flowers using supercritical carbon dioxide extraction (SFE-CO_2_) and to assess the effect of adding 5% ethanol (EtOH) as a co-solvent on extraction yield, in vitro antioxidant capacity in CUPRAC and ABTS assays (TEAC_CUPRAC_ and TEAC_ABTS_), total phenolic (TPC) and flavonoid (TFC) content, β-carotene concentration, and photoprotective potential, expressed as the sun protection factor (SPF). SFE-CO_2_ at 35 MPa and 40 °C resulted in 50% of the total yield within 15 min, with equilibrium reached after 120 min (final yield of 4.6 g/100 g flowers). Co-solvent addition increased yield by ~50% and shortened extraction time. The EtOH-modified extract exhibited markedly higher antioxidant activity, with a 2-fold increase in TEAC_CUPRAC_ (167 mg TE/g E), an 11-fold increase in TEAC_ABTS_ (194 mg TE/g E), and a 3-fold increase in TPC (91 mg GAE/g E), along with improved recovery of flavonoids and β-carotene. Volatile profiling revealed monoterpenoids, aldehydes, and esters as dominant groups, with carvone (14.0–16.5%) and dill ether (4.2–5.8%) as major contributors to aroma. The SFE-CO_2_ + 5% EtOH extract achieved the highest SPF value (49.5 at 1 mg/mL; SPF > 6 at >0.1 mg/mL), indicating strong photoprotective potential and potential suitability for natural antioxidant and cosmetic applications.

## 1. Introduction

*Taraxacum officinale* (common name dandelion), a member of the Asteraceae family, is a perennial flowering plant that grows in moist soils and is believed to have originated in Europe, but is now distributed across the Northern Hemisphere [1]. Historically, many parts of this edible plant have been consumed in various forms. For example, dandelion roots can be roasted and added to coffee, dandelion leaves can be eaten raw in a salad, and dandelion extracts can provide flavour to various products [2]. Moreover, the various parts of this plant have found multilateral applications in traditional and folk medicinal practices [3]. In fact, the rich phytochemistry of this plant material, which includes polyphenols, flavonoids, phytosterols, polysaccharides, sesquiterpenoids, and carotenoids, among other substances, has been highlighted in several recent reviews [4,5,6]. The biological effects of this vast array of phytochemicals are actively being explored in various areas of human health, with many reviews highlighting their antioxidant [7], anti-inflammatory, antimicrobial, wound-healing [8], or gastrointestinal-protective [8] properties.

Interestingly, several studies have demonstrated that dandelion flowers are a potentially exploitable source of natural antioxidants [9,10,11]. Although many studies have employed conventional (i.e., maceration, Soxhlet, decoction) extraction methods to isolate bioactive compounds from various anatomical parts of *T. officinale*, studies on intensifying technologies have mainly focused on ultrasound-assisted extraction, enzyme-assisted extraction, or their combination, and to a lesser extent, supercritical fluid extraction (SFE) [12,13]. Moreover, most available studies have focused mainly on polar (aqueous or ethanol-based) extracts from leaves and roots, largely overlooking the lipophilic fraction in flowers. Currently, CO_2_ is the most widely used supercritical fluid, primarily because it can attain the supercritical state at relatively low pressure (>7.38 MPa) and temperature (>31.1 °C). Additionally, due to its non-toxic and non-flammable nature, low cost, and GRAS status, SFE-CO_2_ has become a widely adopted sustainable extraction method for extracting biologically active lipophilic fractions from a variety of plant materials and their by-products, offering a cleaner and more environmentally friendly option than conventional solid–liquid extraction techniques with hydrocarbon solvents [14,15]. In addition, by minimising the use of organic solvents, avoiding the need to remove toxic solvent residues, and allowing selective recovery of thermolabile compounds through proper selection of pressure and temperature, SFE-CO_2_ is particularly well suited for applications in the food, nutraceutical, and pharmaceutical sectors [16]. Furthermore, the addition of polar co-solvents (e.g., ethanol, water) to neat CO_2_ can further aid in isolating compounds of higher polarity [17]. Despite the interest and potential advantages of this technique, the literature is somewhat limited. Specifically, current research is limited to four recent publications that focus on the extraction and characterisation of *T. officinale* seeds [18,19,20], supplemented by two additional studies, one using dandelion leaves [21] and another with flowers [22].

Given the growing demand for sustainable, plant-derived ingredients for food, nutraceutical, and cosmetic applications, the present study was aimed to isolate lipophilic constituents from *T. officinale* flowers using SFE-CO_2_, with and without ethanol as a co-solvent, and to characterise the resulting extracts in terms of extraction yield, selected phytochemical indices (total phenolic, total flavonoid, and β-carotene content), volatile compound profile, and in vitro antioxidant capacity. In addition to in vitro antioxidant capacity, the photoprotective potential of plant-derived extracts, commonly expressed as the sun protection factor (SPF), is increasingly relevant for ingredients intended for phytodermatological and cosmetic formulations. SPF serves as an in vitro indicator of the material’s ability to attenuate UV radiation and can therefore be used to identify extracts with potential as natural adjuncts or boosters in sunscreen products [23]. Therefore, the photoprotective potential of the SFE-CO_2_ extracts was evaluated as well by determining SPF values at various concentrations. To the best of our knowledge, this is the first comprehensive study to integrate in vitro antioxidant and photoprotective evaluation with analysis of volatile compound composition in lipophilic dandelion flower extracts obtained by SFE-CO_2_.

## 2. Results and Discussion

### 2.1. Preparation of SFE-CO_2_ Extracts from T. officinale Flowers

SFE-CO_2_ is one of the widely used intensifying extraction methods that uses supercritical CO_2_ to isolate lipids, volatile oils, and other non-polar constituents from various matrices [24,25]. Previously, SFE-CO_2_ has been used to extract β-amyrin and β-sitosterol from *T. officinale* leaves [21], and to separate various bioactive compounds from the seeds [18,19,20]. However, information remains scarce on the application of SFE-CO_2_ to *T. officinale* flowers and on the characterisation of the resulting extracts.

**Figure 1:**
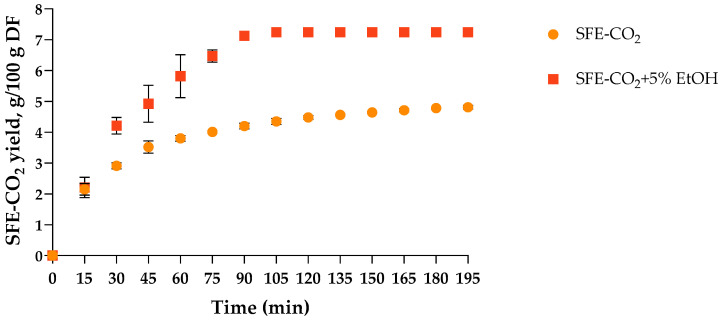
Kinetics of SFE-CO_2_ extraction of *T. officinale* flowers with and without EtOH as co-solvent. DF: *T. officinale* flowers; SFE-CO_2_: supercritical carbon dioxide extraction. Results are expressed as mean ± SD (*n* = 3).

In this study, SFE-CO_2_ was performed at 35 MPa and 40 °C, conditions commonly reported as optimal for the recovery of thermolabile compounds from seeds and leaves of *T. officinale* [18,19,20,21] and from the flowers of other Asteraceae plant species [22]. These parameters ensure maintaining an adequate CO_2_ density (934.9 kg/m^3^) for solubilising non-polar compounds while minimising the risk of thermal degradation. The SFE-CO_2_ kinetics of *T. officinale* flowers with and without co-solvent addition (Figure 1) demonstrated the characteristic three-stage profile typical of supercritical CO_2_ extraction of plant materials, which transitions sequentially through an initial rapid extraction phase, a falling extraction rate, and finally a diffusion-controlled equilibrium [26]. For neat CO_2_, approximately 50% of the final yield was obtained within the first 15 min of the constant extraction-rate period, reflecting the rapid removal of readily accessible, surface-exposed lipophilic compounds such as essential oils and waxes. This was followed by a falling extraction-rate phase, during which approximately 80% of the total yield was reached within 60 min, and subsequently by a diffusion-controlled stage in which equilibrium was reached after 120 min, resulting in a final yield of 4.8 g per 100 g of dandelion flowers (DF) (Figure 1). Incorporating 5% (*v*/*v*) EtOH as a co-solvent increased solvent polarity, improved recovery of moderately polar compounds, and reduced matrix–solute interactions, thereby shortening the extraction process with equilibrium reached after 90 min (Figure 1) and increasing the final yield by ~50% to 7.2 g/100 g DF. For the yield comparison purposes, Soxhlet extraction was performed with hexane, amounting to 6.6 g/100 g DF after 6 h, further highlighting the efficiency and time-saving advantages of SFE-CO_2_, particularly when combined with a polar co-solvent.

Previously, the effectiveness of SFE-CO_2_ in isolating valuable non-polar *T. officinale* constituents has been demonstrated mainly in studies on seeds and leaves. For example, Milovanović et al. observed that SFE-CO_2_ extraction of dandelion seeds at 30–45 MPa yielded 7.4–25.2% depending on the pressure. In contrast, conventional solvent extraction typically requires longer times and higher solvent consumption to achieve similar yields [20]. Simándi et al. reported that SFE-CO_2_ applied to dandelion leaves across a wide range of pressures (15–45 MPa) and temperatures (35–65 °C) produced high-quality extracts (yields of 1.6–3.4%) with minimal thermal degradation and strong selectivity toward β-amyrin and β-sitosterol, outperforming Soxhlet extraction in both selectivity and environmental impact [21]. To the best of our knowledge, the only available study in which SFE-CO_2_ was applied to dandelion flowers (at 20 MPa and 50 °C) did not report extract yields [22], and no previous work has examined the extraction kinetics or yields of SFE-CO_2_, with or without EtOH as a co-solvent, for dandelion flowers. Beyond yield, SFE offers additional advantages such as shorter extraction times due to the high diffusivity and low viscosity of supercritical CO_2_. Moreover, the selectivity and tunability of CO_2_ as a solvent enable targeted recovery of lipophilic compounds by adjusting pressure and temperature. Lastly, SFE-CO_2_ offers multiple environmental benefits, including being non-toxic, non-flammable, and not leaving solvent residues or requiring post-processing steps. Overall, these findings confirm that SFE-CO_2_, particularly when combined with a polar co-solvent, is a sustainable and efficient alternative to conventional extraction methods for isolating bioactive lipophilic fractions from plant matrices.

### 2.2. In Vitro Antioxidant and Photoprotective Properties of T. officinale SFE-CO_2_ Extracts

Evaluating the bioactive properties of plant-derived extracts is crucial for a deeper understanding of their potential applications in health and cosmetic formulations. In this part of the study, the SFE extracts of *T. officinale* flowers were assessed for their in vitro antioxidant capacity using CUPRAC and ABTS assays, total phenolic content (TPC), total flavonoid content (TFC), and *β*-carotene concentration (Table 1). The specific phytochemical markers are closely associated with the extract’s ability to neutralise free radicals and contribute to photoprotection. Additionally, the sun protection factor (SPF) of the extracts was determined (Table 2) to explore their potential as natural UV filters.

Co-solvent addition substantially increased the antioxidant capacity of the dandelion flower extracts in comparison to neat SFE-CO_2_. On an extract mass basis, the TEAC_CUPRAC_ value nearly doubled from 84.4 to 169.8 mg TE, whereas the TEAC_ABTS_ value augmented ~11-fold (from 17.5 to 193.8 mg TE), indicating a substantial enhancement in overall antioxidant potential (ABTS radical scavenging and CUPRAC reducing capacity). This increase is in agreement with the 1.6- to 3-fold higher carotenoid content (from 28.2 to 44.7 mg/g E), TFC (from 13.1 to 23.9 mg QE/g E), and TPC (from 29.1 to 91.3 mg GAE/g E) values. Given the higher SFE-CO_2_ + 5% EtOH extraction yield, these results translated into proportionally greater values when expressed on a per-mass basis of the plant material (Table 1). The observed assay-dependent improvements in the in vitro antioxidant capacity values are consistent with the methods’ chemistry and the anticipated composition of the extracts. In the CUPRAC assay, antioxidant capacity is quantified as the sample’s overall reducing power, based on its ability to reduce Cu(II) to Cu(I) and thereby form the coloured Cu(I)–neocuproine complex. In contrast, the ABTS assay quantifies the sample’s ability to scavenge the ABTS radical cation, measured as a decrease in absorbance at ~734 nm, and proceeds via electron transfer and/or hydrogen atom transfer mechanisms. In both ABTS and CUPRAC assays, the responses in terms of TEAC values generally increase with the concentration and redox potential of phenolic/flavonoid antioxidants [27,28]. Additionally, CUPRAC performs well at near-neutral pH and can detect both hydrophilic and lipophilic antioxidants, whereas ABTS^•+^ is particularly sensitive to phenolics and conjugated systems that can delocalise charge. Thus, the stronger ABTS response (11-fold per extract basis) is in line with the 3-fold increase in TPC and the nearly 2-fold increase in TFC after co-solvent addition. Introducing a small fraction of EtOH enhances the polarity and hydrogen-bonding capacity of the supercritical phase, thereby improving the solubility and mass transfer of moderately polar phenolics and flavonoids that are otherwise poorly extracted by neat CO_2_, with a solvating behaviour resembling that of hexane. In addition, the greater increase in TEAC_ABTS_ compared with TEAC_CUPRAC_ may be attributed to the carotenoid content, since these phytochemicals predominantly act as radical quenchers rather than strong reducing agents.

The findings of this study are consistent with a previous report by Milovanovic et al., in which the authors defatted dandelion seeds using neat SFE-CO_2_ and subsequently performed a second extraction on the defatted material with EtOH as a co-solvent. They reported that the TPC and TFC of the EtOH-derived extract increased by 4-fold and 5-fold, respectively, while the carotenoid content remained unchanged [18]. Overall, the TPC and TFC values of the tested dandelion flower extracts were above previously reported ranges for SFE-CO_2_ seed extracts (TPC 5.5–17.1 mg GAE/g E; TFC 0.2–1.3 mg QE/g E) [18,20]. Higher phenolic and flavonoid content in flowers compared with seeds is expected [9,10,11] and is consistent with recently published LC–MS/MS data reporting a total of 4.7–4.9 mg of flavonoids (glycosides, aglycones, biflavones, and flavonolignans) per gram of flowers [29]. Carotenoid levels in *Taraxacum* species can vary considerably depending on the species, plant part analysed, geographic location, climatic conditions, and other factors [9,10,11]. For instance, carotenoid contents reported for *T. officinale* leaves ranged from 0.1 mg/g DW in samples collected in Brazil [30] to 0.9 mg/g in those obtained from Poland [31]. Relatively high levels (~1 mg/g dried plant material), comparable to those obtained in this study, have also been reported for Mongolian dandelion (*T. formosanum*) [32]. Overall, higher carotenoid levels in flowers could be anticipated, as their intense yellow pigmentation is strongly associated with carotenoid accumulation.

Despite the documented in vitro antioxidant capacity of polar dandelion extracts [9,10,11], information on the activity of non-polar fractions from aerial parts other than seeds, particularly flowers, remains very limited. For example, Hu and Kits reported that the ethyl acetate fraction of dandelion flower extract exhibited higher antioxidant activity (94% scavenging at 53 μg/mL) in the stable DPPH radical model compared to the water fraction (52% scavenging at 53 μg/mL). Both fractions also protected supercoiled DNA from damage caused by site-specific and non-site-specific hydroxyl radicals [33].

**Figure 2:**
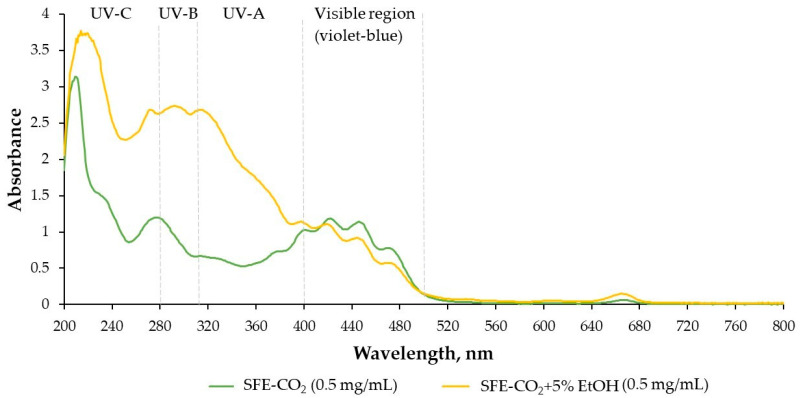
UV–Vis spectra of *T. officinale* SFE-CO_2_ and SFE-CO_2_ + 5% EtOH extracts at 0.5 mg/mL concentration.

The UV–Vis spectra of *T. officinale* SFE-CO_2_ and SFE-CO_2_ + 5% EtOH extracts (Figure 2) show distinct absorption patterns across the UV and visible ranges, indicating differences in their potential photoprotective properties. The addition of EtOH as a co-solvent produced extracts with much higher absorbance capacity in the UV-C (200–280 nm), UV-B (280–315 nm), and UV-A (315–400 nm) regions, which is consistent with their substantially higher TPC, TFC, and enhanced in vitro antioxidant activity (Table 1). In contrast, the neat SFE-CO_2_ extract displayed lower UV absorbance, but showed comparatively high intensity in the visible violet-blue region (400–480 nm), contributing to visible-light filtering properties. The photoprotective potential, expressed as SPF values, also showed an apparent concentration-dependent increase (Table 2). For the SFE-CO_2_ + 5% EtOH extract, SPF rose from ~3.0 at 0.05 mg/mL to 49.5 at 1.00 mg/mL, with corresponding UV-B absorption increasing from 66% to 98%. In comparison, the neat SFE-CO_2_ extract showed a more modest increase, with SPF values rising from 0.8 to 13.9 over the same concentration range, reaching 93% UVB absorption at 1.00 mg/mL (Table 2).

Although in vitro SPF values cannot be directly equated with in vivo ISO standards, the Mansur method is widely accepted for preliminary screening and aligns reasonably well with standardised SPF concepts. This method evaluates weighted absorbance in the 290–320 nm range, where erythemally effective UV-B radiation is most intense [34]. According to the EU Commission Recommendation 2006/647/EC, sunscreen efficacy is categorised into four levels: low (SPF 6–10), medium (SPF 15–25), high (SPF 30–50), and very high (SPF > 50) [35]. Using this framework for qualitative context, the SFE-CO_2_ + 5% EtOH extract at 1.00 mg/mL (SPF of 49.5) falls within the upper range of the “high” category, approaching the “very high” threshold (>50), while the neat SFE-CO_2_ extract provides low to moderate protection across the tested concentrations. The higher TPC, TFC, and carotenoid levels in the EtOH-modified SFE-CO_2_ extract provide a plausible explanation for its significantly higher SPF values and UV-B absorption potential. Also, the compositional factors driving SPF performance are consistent with trends in the antioxidant assays. Flavonoids, which absorb in the near UV spectrum (bands I–II; 300–400 nm), contribute both direct UV filtering and indirect antioxidant protection, while carotenoids primarily act as singlet oxygen and radical quenchers, absorbing strongly in the visible range, and complement activity of phenolics by mitigating oxidative cascades triggered by UV exposure [36,37,38,39], also acting as anti-inflammatory agents that help reduce photodamage [40]. Therefore, future studies should prioritise targeted profiling and quantification of individual phenolic acids and flavonoids to establish more precise structure–activity relationships leading to the photoprotective effects of *T. officinale* extracts.

It is important to note that data on the skincare effects of *T. officinale* extracts, particularly their UV-protective properties, remain very limited, with only a few reports confirming polar-fraction activity. For example, Yang and Li showed that water extracts from dandelion leaves and flowers, but not roots, could effectively protect human dermal fibroblasts from UV-B-induced damage and hydrogen peroxide-induced oxidative stress, primarily by reducing reactive oxygen species generation and inhibiting matrix metalloproteinase activity [41]. In addition, among six extracts prepared from *T. officinale* stems and leaves using ultrasound-assisted extraction with H_2_O, EtOH, and EtOH/H_2_O mixture (50%, *v*/*v*), the hydroethanolic stem extract showed the strongest UV-A and UV-B absorption at 10 mg/mL concentration, although its effectiveness was lower than that of chlorogenic acid [42]. To the best of our knowledge, this is the first report on the in vitro photoprotective activity of non-polar dandelion flower extracts.

### 2.3. Volatile Compound Profile of T. officinale Flowers and SFE-CO_2_ Extracts

**Figure 3:**
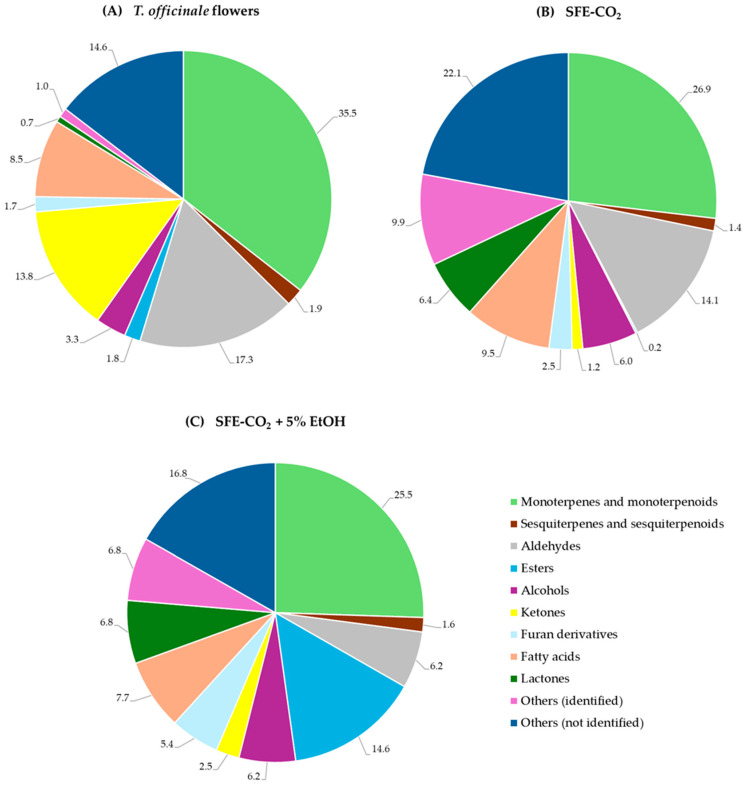
The composition (% of the total GC peak area) of volatile compound groups in the headspace of *T. officinale* flowers (**A**), SFE-CO_2_ (**B**), and SFE-CO_2_ + 5% EtOH (**C**) extracts. Percentages are displayed rounded to the first decimal place (the corresponding unrounded values sum to 100%).

SPME-GC × GC-TOF-MS was employed to compare the composition of volatile compounds in *T. officinale* flowers and their SFE extracts obtained using neat CO_2_ and CO_2_ modified with 5% EtOH, indicating significant qualitative and quantitative differences (Figure 3). Headspace SPME is an equilibrium-based, non-exhaustive technique with significant matrix and analyte-dependent partitioning, which has severe limitations concerning the overall quantification of analytes, as previously highlighted in the literature. As a result, volatile data in this study are reported semi-quantitatively as percentages of the total GC peak area for comparative purposes across samples. No internal or external standards were employed, consistent with reported challenges of surrogate normalization under HS-SPME conditions in complex matrices [43,44,45,46]. The total GC peak area, representing the cumulative abundance of volatile compounds detected in the sample headspace, was highest for the flowers and nearly equivalent for the SFE-CO_2_ + 5% EtOH extract. In contrast, the neat CO_2_ extract showed an approximately 26% lower total area (Table 3). A total of 69 volatile compounds, categorised into ten chemical groups (Figure 3), were identified across the *T. officinale* flowers, SFE-CO_2_, and SFE-CO_2_ + 5% EtOH extracts, including 13 monoterpenes and monoterpenoids, 4 sesquiterpenes and sesquiterpenoids, 11 aldehydes, 4 esters, 4 alcohols, 10 ketones, 4 furan derivatives, 5 fatty acids, 5 lactones, and 9 other identified compounds. A substantial portion of the volatile profile was identified, averaging ~82% across all samples, with the unidentified volatile content averaging ~18% of the total GC peak area (Figure 3). Notably, several of these identified volatiles are known for their roles in plant defence, aroma, and pharmacological properties, indicating the value of dandelion and its extracts as potential sources of functional metabolites [10,11].

**Figure 4:**
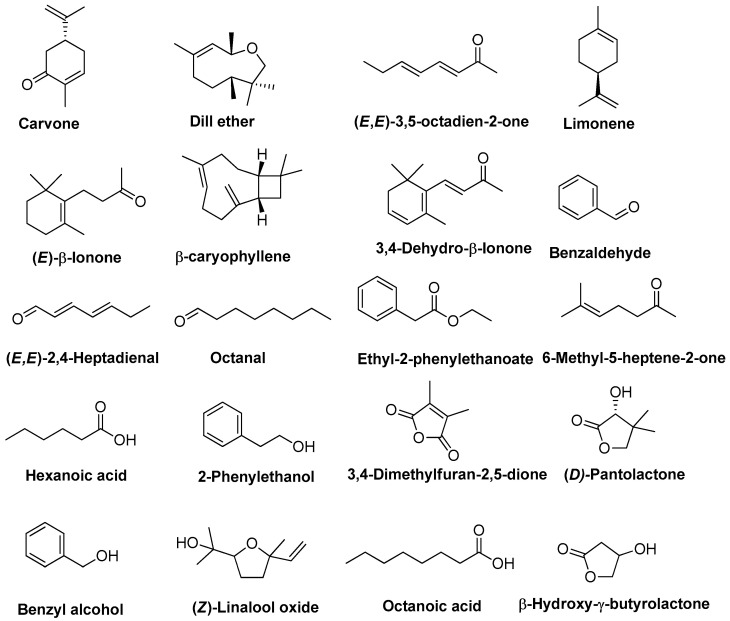
Structures of major volatiles identified in *T. officinale* flowers, SFE-CO_2_ and SFE-CO_2_ + 5% EtOH extracts.

As presented in Figure 3, monoterpenes and monoterpenoids represented the most significant fraction of volatiles, accounting for 35.5% in flowers, 26.9% in SFE-CO_2_, and 25.5% in SFE-CO_2_ + 5% EtOH of the total quantified by GC volatiles. Within this class of volatiles, carvone (Figure 4) was the most abundant compound across all three samples. The distinguishing between the two carvone enantiomers cannot be achieved by a standard GC–MS analysis without a chiral column; however, some reports indicate that sweet, spearmint-like, herbal, minty odour-imparting (−)-carvone is more common for aromatic and medicinal plants of the Lamiaceae and Asteraceae families, whereas (+)-carvone is more characteristic of Apiaceae species [47]. As reported in Table 3, its headspace concentration decreased slightly in the SFE extracts (~14%) compared to *T. officinale* flowers (16.5%). Similar trends were observed for the dill ether (Figure 4), averaging 4.4% in the headspace of SFE extracts, compared with 5.8% in dried flowers. (*E)*-Dihydrocarvone and estragole exhibited nearly identical behaviour during SFE, both showing an approximate one-third reduction in headspace concentration as compared to the flowers, which moderated their respective herbal, warm, and anisic and spicy notes in the resulting extracts. As reported in Table 3, SFE generally reduced pine, peppery, spicy, and floral flavour notes in the extracts. Specifically, limonene (Figure 4), one of the major headspace components in the flowers (3.8%), was not detected in the SFE-CO_2_ extract. Although the addition of 5% EtOH allowed for its partial recovery (0.3%), this negligible concentration in the overall volatile profile, coupled with the absence of several other minor monoterpenes (<1% of the total GC peak area), like α-phellandrene, linalool, and β-cyclocitral, indicates a significant reduction in the initial complex terpenic flavour profile of *T. officinale* flowers. Interestingly, (*Z*)-linalool oxide (pyranoid) concentration in the headspace was enriched by the neat CO_2_, increasing nearly 10-fold from a minor constituent in the flowers (0.4%) to a high share of ~4%, partially compensating for the loss of citrus, green, and floral characteristics of the SFE-CO_2_ extract. However, the addition of 5% EtOH as a co-solvent reduced the relative content of this volatile compound to 2.4%, suggesting a change in solubility favouring other polar constituents in the SFE process. Sesquiterpenes and sesquiterpenoids constituted a minor group of volatiles, whose total contribution remained relatively stable across the different samples (Figure 3), ranging from 1.4% (SFE-CO_2_) to 1.9% (flowers), providing woody, spicy, and floral base notes.

The percentage of aldehydes ranged from 6.2 to 17.3% in the headspace of the SFE-CO_2_ + 5% EtOH and flower samples, respectively (Figure 3). Benzaldehyde (Figure 4) was the most abundant aldehyde in the dandelion flowers (8.4%), whose share in the headspace was significantly reduced up to 1% by SFE with or without the addition of polar modifier (Table 3). Benzaldehyde is a key aldehyde identified in multiple monovarietal *T. officinale* honeys [48,49]. Due to its bitter almond and cherry-like odour, it is used in various scent compositions and as a precursor to several aliphatic fragrance and flavouring ingredients [50]. Interestingly, (*E*,*E*)-2,4-heptadienal (Figure 4), which was absent in the flower headspace, was the dominant aldehyde in the neat CO_2_ extract and, combined with (*E*,*Z*)-2,4-heptadienal, amounted to 7.2% of the total GC peak area, imparting a distinct fatty, oily, and vegetable aroma. In contrast, the addition of 5% EtOH as a co-solvent markedly reduced the levels of these fatty dienals, with octanal, nonanal, and decanal being the predominant aldehydes and providing a waxy-citrusy aroma profile to the SFE-CO_2_ + 5% EtOH extract (Table 3). In a previous report, aldehydes including octanal, phenylacetaldehyde, 2-methylbenzaldehyde, nonanal, pentadecanal, and 10-undecenal have also been reported in the essential oil of dandelion [51].

**Table 3:** Headspace volatile compound composition (% of the total GC peak area) of *T. officinale* flowers, SFE-CO_2_ and SFE-CO_2_ + 5% EtOH (C) extracts.

Compound	LRI_exp_	LRI_lit_	Exact Mass	Formula	% of the Total GC Peak Area			Odour Type: Description ^A^
					Flowers	SFE-CO_2_	SFE-CO_2_ + 5% EtOH	
Monoterpenes and monoterpenoids								
α-Phellandrene	1006	1007 [52]	136.1252	C_10_H_16_	0.84 ± 0.03	-^ND^	-^ND^	Terpenic: citrus, herbal, terpenic, green, woody, black pepper
*p*-Cymene	1027	1020 [53]	134.1096	C_10_H_14_	0.43 ± 0.01	-^ND^	-^ND^	Terpenic: citrus, sweet
Limonene	1030	1024 [53]	136.1252	C_10_H_16_	3.78 ± 0.23 ^b^	-^ND^	0.33 ± 0.10 ^a^	Terpenic: terpenic, pine, peppery
Linalool	1102	1095 [53]	154.1358	C_10_H_18_O	0.35 ± 0.02	-^ND^	-^ND^	Floral: citrus, orange, floral, waxy, rose
(*Z*)-Linalool oxide (pyranoid)	1175	1170 [53]	170.1307	C_10_H_18_O_2_	0.44 ±0.19 ^a^	3.97 ± 0.00 ^c^	2.36 ± 0.00 ^b^	Citrus: citrus, green
Dill ether	1193	1184 [53]	152.1201	C_10_H_16_O	5.83 ± 0.02 ^c^	4.26 ± 0.00 ^a^	4.54 ± 0.00 ^b^	Herbal: herbal, dill
(*E*)-Dihydrocarvone	1203	1200 [53]	152.1201	C_10_H_16_O	2.28 ±0.05 ^b^	1.47 ± 0.13 ^a^	1.50 ± 0.05 ^a^	Herbal: warm, herbal
Estragole	1205	1195 [53]	148.0888	C_10_H_12_O	2.19 ± 0.07 ^b^	1.47 ± 0.13 ^a^	1.50 ± 0.05 ^a^	Anisic: sweet, phenolic, anise, spicy, green, herbal, minty
Safranal	1207	1196 [53]	150.1045	C_10_H_14_O	1.51 ± 0.19 ^b^	-^ND^	0.24 ± 0.05 ^a^	Herbal: fresh, herbal, phenolic, metallic, rosemary, tobacco, spicy
β-Cyclocitral	1228	1217 [53]	152.1201	C_10_H_16_O	0.54 ± 0.07	-^ND^	-^ND^	Tropical: tropical, saffron, herbal, rose, sweet, tobacco, green, fruity
Carvone	1253	1242 [53]	150.1045	C_10_H_14_O	16.46 ± 0.10 ^b^	14.10 ±0.17 ^a^	14.03 ± 0.06 ^a^	Herbal: spicy, green, sweet, spearmint, mint, carraway dill
Thymol	1296	1289 [53]	150.1045	C_10_H_14_O	0.16 ± 0.04 ^a^	0.91 ± 0.01 ^c^	0.56 ± 0.07 ^b^	Herbal: herbal, thyme, phenolic, medicinal, camphoreous
(*E*)-Geranyl acetone	1457	1453 [53]	194.1671	C_13_H_22_O	0.73 ± 0.01 ^b^	0.69 ± 0.04 ^b^	0.46 ± 0.06 ^a^	Floral: fresh, green, fruity, waxy, rose, woody, magnolia, tropical
Sesquiterpenes and sesquiterpenoids								
β-Caryophyllene	1429	1420 [52]	204.1878	C_15_H_24_	0.62 ± 0.13 ^a^	0.64 ± 0.23 ^a^	0.44 ± 0.07 ^a^	Woody: woody, spicy
(*E*)-β-Farnesene	1460	1459 [52]	204.1878	C_15_H_24_	0.33 ± 0.00 ^b^	0.04 ± 0.00 ^a^	-^ND^	Woody: woody, citrus, herbal, sweet
3,4-Dehydro-β-ionone	1492	1485 [54]	190.1358	C_13_H_18_O	0.12 ± 0.00 ^a^	0.33 ± 0.00 ^b^	0.57 ± 0.00 ^c^	Floral: sweet, floral, fruity, woody
(*E*)-β-Ionone	1494	1487 [53]	192.1514	C_13_H_20_O	0.85 ± 0.02 ^b^	0.36 ± 0.11 ^a^	0.54 ± 0.08 ^a^	Floral: sweet, floral, fruity, woody
Aldehydes								
(*Z*)-2-Heptenal	956	947 [53]	112.0888	C_7_H_12_O	0.41 ± 0.03 ^a^	1.33 ± 0.21 ^b^	-^ND^	Green: green, fatty
Benzaldehyde	962	962 [52]	106.0419	C_7_H_6_O	8.44 ± 0.18 ^c^	1.02 ± 0.05 ^b^	0.72 ± 0.08 ^a^	Fruity: sweet, bitter, almond, cherry
(*E,Z*)-2,4-Heptadienal	999	990 [52]	110.0732	C_7_H_10_O	0.68 ± 0.00 ^a^	2.60 ± 0.08 ^b^	-^ND^	Green: green, pungent, fruity, spicy
Octanal	1004	998 [53]	128.1201	C_8_H_16_O	3.68 ± 0.21 ^b^	1.89 ± 0.00 ^a^	1.67 ± 0.00 ^a^	Aldehydic: aldehydic, waxy, citrus, orange peel, green, herbal, fresh, fatty
(*E,E*)-2,4-Heptadienal	1013	1005 [53]	110.0732	C_7_H_10_O	-^ND^	4.62 ± 0.20 ^b^	0.23 ± 0.03 ^a^	Fatty: fatty, green, oily, aldehydic
Phenylacetaldehyde	1048	1036 [53]	120.0575	C_8_H_8_O	0.58 ± 0.21 ^b^	0.08 ± 0.00 ^a^	0.84 ± 0.00 ^b^	Green: green, sweet, floral, hyacinth, clover, honey, cocoa
(*E*)-2-Octenal	1060	1049 [53]	126.1045	C_8_H_14_O	0.36 ± 0.16	-^ND^	-^ND^	Fatty: fresh, cucumber, fatty, green, herbal, banana, waxy, green leafy
Nonanal	1106	1103 [52]	142.1358	C_9_H_18_O	1.14 ± 0.13 ^a^	1.19 ± 0.11 ^a^	1.35 ± 0.14 ^a^	Aldehydic: waxy, aldehydic, rose, fresh, orris, orange peel, fatty, citrus
2,4-Dimethylbenzaldehyde	1181	1180 [55]	134.0732	C_9_H_10_O	0.33 ± 0.07 ^a^	-^ND^	0.30 ± 0.00 ^a^	Naphthyl: naphthyl, cherry, almond
Decanal	1209	1206 [52]	156.1514	C_10_H_20_O	1.65 ± 0.19 ^c^	0.47 ± 0.12 ^a^	1.04 ± 0.07 ^b^	Aldehydic: sweet, aldehydic, waxy, orange peel, citrus, floral
*p*-Anisaldehyde	1264	1247 [53]	136.0524	C_8_H_8_O_2_	-^ND^	0.87 ± 0.03	-^ND^	Anisic: sweet, powdery, vanilla, anise, woody, coumarinic, creamy, spicy
Esters								
Methyl octanoate	1126	1123 [53]	158.1307	C_9_H_18_O_2_	0.96 ± 0.05	-^ND^	-^ND^	Waxy: waxy, green, sweet, orange, aldehydic, vegetable, herbal
Ethyl octanoate	1200	1196 [53]	172.1463	C_10_H_20_O_2_	0.32 ± 0.04 ^a^	-^ND^	0.38 ± 0.02 ^a^	Waxy: fruity, winey, waxy, sweet, apricot, banana, brandy, pear
Ethyl 2-phenylethanoate	1252	1243 [53]	164.0837	C_10_H_12_O_2_	-^ND^	-^ND^	14.03 ± 0.00	Floral: sweet, floral, honey, rose
Methyl dodecanoate	1527	1524 [53]	214.1933	C_13_H_26_O_2_	0.47 ± 0.00 ^b^	0.18 ± 0.01 ^a^	0.17 ± 0.04 ^a^	Waxy: waxy, soapy, creamy, coconut
Alcohols								
Heptan-1-ol	970	959 [53]	116.1201	C_7_H_16_O	0.75 ± 0.00	-^ND^	-^ND^	Green: musty, pungent, leafy, green, vegetable, fruity, apple, banana
Oct-1-en-3-ol	979	974 [53]	128.1201	C_8_H_16_O	1.07 ± 0.10 ^b^	0.11 ± 0.00 ^a^	0.10 ± 0.03 ^a^	Earthy: earthy, green, oily, vegetable
Benzyl alcohol	1039	1026 [53]	108.0575	C_7_H_8_O	0.29 ± 0.00 ^a^	2.05 ± 0.34 ^b^	2.22 ± 0.09 ^b^	Floral: sweet, floral, fruity, chemical
2-Phenylethanol	1119	1116 [54]	122.0732	C_8_H_10_O	1.22 ± 0.00 ^a^	3.80 ± 0.23 ^b^	3.83 ± 0.00 ^b^	Floral: sweet, floral, fresh, rose, honey
Ketones								
3-Octanone	987	979 [53]	128.1201	C_8_H_16_O	2.72 ± 0.00	-^ND^	-^ND^	Herbal: herbal lavender sweet mushroom, fermented, green, vegetable
6-Methyl-5-heptene-2-one	988	981 [53]	126.1045	C_8_H_14_O	2.72 ± 0.00 ^c^	0.84 ± 0.04 ^b^	0.42 ± 0.03 ^a^	Citrus: fruity, apple, musty, ketonic, creamy, cheesy, banana
2-Octanone	992	988 [53]	128.1201	C_8_H_16_O	0.38 ± 0.14	-^ND^	-^ND^	Earthy: earthy, weedy, natural, woody, herbal, dairy
3-Octen-2-one	1041	1030 [53]	126.1045	C_8_H_14_O	0.61 ± 0.12	-^ND^	-^ND^	Earthy: earthy, spicy, herbal, sweet, mushroom, hay, blueberry
(*E,E*)-3,5-octadien-2-one	1074	1068 [56]	124.0888	C_8_H_12_O	3.78 ± 0.11 ^b^	-^ND^	0.65 ± 0.05 ^a^	Fruity: fruity, green, grassy
2-Nonanone	1094	1087 [53]	142.1358	C_9_H_18_O	0.73 ± 0.01	-^ND^	-^ND^	Fruity: fresh, sweet, green, herbal
3,5-Octadien-2-one	1096	1102 [57]	124.0888	C_8_H_12_O	0.80 ± 0.16 ^b^	-^ND^	0.22 ± 0.00 ^a^	Fatty: fruity, fatty, mushroom
6-Methyl-3,5-heptadien-2-one	1109	1105 [58]	124.0888	C_8_H_12_O	0.81 ± 0.08 ^b^	-^ND^	0.24 ± 0.06 ^a^	Spicy: green, spicy, cooling, herbal
4-Ketoisophorone	1149	1140 [53]	152.0837	C_9_H_12_O_2_	1.00 ± 0.03 ^b^	-^ND^	0.42 ± 0.05 ^a^	Musty: woody, sweet, tea, tobacco, leafy, citrus, lemon
Hexahydrofarnesyl acetone	1845	1836 ^B^	268.2766	C_13_H_36_O	0.25 ± 0.04 ^a^	0.31± 0.09 ^a^	0.59 ± 0.00 ^b^	Woody: woody, floral, jasmine, green
Furan derivatives								
2-Pentylfuran	992	984 [53]	138.1045	C_9_H_14_O	0.35 ± 0.06 ^b^	0.12 ± 0.02 ^a^	0.12 ± 0.00 ^a^	Fruity: fruity, green, earthy, beany, vegetable, metallic
3,4-Dimethylfuran-2,5-dione	1043	1038 [59]	126.0317	C_6_H_6_O_3_	-^ND^	2.39 ± 0.00 ^a^	3.17 ± 0.00 ^b^	
2,3-Dihydrobenzofuran	1226	1226 [60]	120.0575	C_8_H_8_O	-^ND^	-^ND^	0.92 ± 0.06	
Dihydroactinidiolide	1547	1539 [61]	180.115	C_11_H_16_O_2_	1.30 ±0.01 ^b^	-^ND^	1.14 ± 0.07 ^a^	Fruity: ripe apricot, fruity, plum, berry, grape, fruit, tropical fruit, woody
Fatty acids								
Hexanoic acid	1012	1020 [62]	116.0837	C_6_H_12_O_2_	1.92 ± 0.15 ^b^	4.64 ± 0.00 ^c^	1.43 ± 0.15 ^a^	Fatty: sour, fatty, sweaty, cheesy
Heptanoic acid	1092	1097 [63]	130.0994	C_7_H_14_O_2_	0.23 ± 0.00 ^a^	0.94 ± 0.00 ^c^	0.56 ± 0.00 ^b^	Cheesy: waxy, fermented, fruity
Octanoic acid	1194	1179 [62]	144.115	C_8_H_16_O_2_	5.99 ± 0.23 ^c^	2.18 ± 0.00 ^a^	4.32 ± 0.00 ^b^	Fatty: fatty, waxy, rancid, oily, vegetable, cheesy
Nonanoic acid	1276	1273 [48]	158.1307	C_9_H_18_O_2_	0.33 ± 0.04 ^a^	0.53 ± 0.00 ^b^	0.30 ± 0.08 ^a^	Waxy: waxy, cheesy, dairy
Dodecanoic acid	1569	1565 [53]	186.162	C_12_H_24_O_2_	-^ND^	1.16 ± 0.00 ^b^	1.06 ±0.00 ^a^	Fatty: fatty, coconut, bay
Lactones								
γ-Valerolactone	957	958 [64]	100.0524	C_5_H_8_O_2_	0.30 ± 0.11 ^a^	0.91 ± 0.00 ^c^	0.60 ± 0.02 ^b^	Herbal: herbal, warm, tobacco, woody
d-Pantolactone	1042	1032 ^B^	130.0630	C_6_H_10_O_3_	-^ND^	2.18 ± 0.11 ^a^	3.17 ± 0.00 ^b^	
γ-Caprolactone	1060	1062 [64]	114.0681	C_6_H_10_O_2_	0.36 ± 0.16 ^a^	-^ND^	0.68 ± 0.00 ^b^	Tonka: coconut, sweet, tobacco
β-Hydroxy-γ-butyrolactone	1180	1185 ^B^	102.0317	C_4_H_6_O_3_	-^ND^	2.89 ± 0.00 ^a^	2.36 ± 0.00 ^b^	
γ-Nonalactone	1369	1363 [65]	156.1150	C_9_H_16_O_2_	-^ND^	0.44 ± 0.00	-^ND^	Coconut: coconut, creamy, waxy, sweet, buttery, oily
Others (identified)								
Maltol	1121	1110 [66]	126.0317	C_6_H_6_O_3_	-^ND^	1.03 ± 0.00 ^b^	0.63 ± 0.00 ^a^	Caramellic: sweet, caramellic, cotton candy, jammy, fruity, baked bread
*N*-Formylmorpholine	1135	1133 [67]	115.0633	C_5_H_9_NO_2_	-^ND^	1.56 ± 0.17	-^ND^	Mild
Benzeneacetonitrile	1146	1134 [53]	117.0578	C_8_H_7_N	0.67 ± 0.16 ^a^	0.58 ± 0.00 ^a^	1.57 ± 0.00 ^b^	
2,3-Dihydro-3,5-dihydroxy-6-methyl-4h-pyran-4-one	1150	1140 [68]	144.0423	C_6_H_8_O_4_	-^ND^	0.28 ± 0.00 ^a^	1.90 ± 0.08 ^b^	
1-Acetylpyrrolidine	1179	1162 ^B^	113.0841	C_6_H_11_NO	0.13 ± 0.01 ^a^	0.47 ± 0.09 ^b^	0.26 ± 0.00 ^a^	
Butyl diglycol	1194	1198 [69]	162.1256	C_8_H_18_O_3_	-^ND^	0.76 ± 0.06 ^a^	1.05 ± 0.00 ^b^	
Benzoic acid	1197	1196 [70]	122.0368	C_7_H_6_O_2_	-^ND^	2.98 ± 0.00 ^b^	1.05 ± 0.00 ^a^	Balsamic: balsamic, urine
Benzenacetic acid	1262	1255 [62]	136.0524	C_8_H_8_O_2_	-^ND^	1.81 ± 0.00 ^b^	0.14 ± 0.00 ^a^	
2,4,6,8-tetramethylundecene	1342	1330 ^B^	210.2348	C_15_H_30_	0.20 ± 0.10 ^a^	0.47 ± 0.00 ^b^	0.24 ± 0.05 ^a^	
Total GC peak area AU × 10^6^					401.65 ± 43.07	294.95 ± 26.18	399.18 ± 28.16	

^A^: Odour descriptions obtained from The Goodscent Company (http://www.thegoodscentscompany.com/; accessed on 8 September 2025) and Olfactorian (https://olfactorian.com/; accessed on 8 September 2025) databases; ^B^: retention index from PubChem database (https://pubchem.ncbi.nlm.nih.gov/; accessed on 4 August 2025); -^ND^—not detected. Results are expressed as mean ± SD (*n* = 3). Different superscript letters in the same column indicate significantly different values (*p* < 0.05) based on a two-tailed unpaired *t*-test or a one-way ANOVA and Tukey’s test.

Among the fatty acids (7.7–9.5% of the total GC peak area across different samples), octanoic acid (Figure 4) accounted for the highest percentage in the dandelion flowers (6.0%) and the SFE-CO_2_ + 5% EtOH extract (4.3%), whereas hexanoic acid (Figure 4) was the most abundant in the neat SFE-CO_2_ extract (4.6%) (Table 3). As shown in Figure 3, the SFE extracts, particularly those obtained with EtOH as a co-solvent, contained markedly higher proportions of esters, alcohols, lactones, and furan derivatives than the dandelion flowers. For example, esters reached 14.6% of headspace volatiles in the SFE-CO_2_ + 5% EtOH extract versus only 0.2% in the neat CO_2_ extract and 1.8% in the flowers (Figure 3), with ethyl 2-phenylethanoate (sweet, floral, honey, rose, balsamic notes) as the major ester in the co-solvent modified extract (Table 3). Jerković et al. reported that ethyl 2-phenylethanoate was also identified in honey produced from *T. officinale* monofloral honeys [48]. The highest percentage of furan derivatives was also found in the SFE-CO_2_ + 5% EtOH sample headspace (5.4%), followed by the neat SFE-CO_2_ extract (2.5%) and flowers (1.7%) (Figure 3). Alcohols (6.1% in SFE extracts vs. 3.3% in flowers) and lactones (6.6% vs. <1%) followed similar trends, with 2-phenylethanol, benzyl alcohol, d-pantolactone, and β-hydroxy-γ-butyrolactone identified as predominant contributors imparting sweet, floral, fresh, honey-like, and fruity notes to the volatile profile of the SFE extracts. Interestingly, ketones were characteristic volatiles of the dandelion flower sample (13.8%), dominated by (*E*,*E*)-3,5-octadien-2-one, 3-octanone, and 6-methyl-5-hepten-2-one, which impart green, grassy, woody, and earthy notes, whereas their presence in the headspace of the SFE extracts was very low (Table 3).

To the best of our knowledge, this is one of the most comprehensive analyses of aroma-active components in dandelion flowers and the plant’s supercritical CO_2_ extracts. Although direct comparisons may not always be feasible, these findings complement and extend the existing knowledge on the volatile profiles of various anatomical parts of dandelion, isolated using a range of extraction techniques and conditions. In the only other study, reporting the volatile profile of SFE extract of dandelion flowers, Schoss et al. reported a total of 11 identified compounds, with heneicosane and phytol contributing ~28% and 8%, respectively, with the extract composition, though remaining largely unidentified, as ~61% of the compounds were not characterised [22]. In another study, Bylka et al. analysed the volatile profile of the essential oil obtained by hydrodistillation from *T. officinale* flowers and reported the identification of 25 volatile constituents [51]. The authors indicated that the major components included 1,3-dimethylbenzene, 1,2-dimethylbenzene, 1-ethyl-3-methylbenzene, heneicosane, and tricosane, indicating a composition rich in aromatic hydrocarbons and long-chain alkanes [51]. In another study, the n-hexane-soluble compounds from dandelion aerial parts were compared across different growth stages. GC-MS analysis identified 30 biologically active substances in the non-polar fraction, with the main components being phytol (14.7%), lupeol (14.5%), taraxasteryl acetate (11.4%), β-sitosterol (10.3%), α-amyrin (9.0%), β-amyrin (8.3%), and cycloartenol acetate (5.8%) [71]. Moreover, Zhang et al. performed a comprehensive analysis of seven dandelion samples, comprising three *T. kok-saghyz* and four *T. officinale* accessions, reporting 105 and 107 volatile compounds in the leaves and roots, respectively [72]. The leaves were characterised by 9 alcohols, 15 aldehydes, 9 acids, 25 esters, 17 ketones, 7 alkenes, 7 aromatic compounds, 4 alkanes, 2 ethers, 3 phenols, 1 furan, 1 pyrazine, and 5 additional compounds. The authors reported that ethyl tetradecanoate, ethyl linolenate, ethyl linoleate, dihydroactinidiolide, ethyl palmitate, β-ionone, 3,5-octadien-2-one, β-ionone 5,6-epoxide, geranyl acetone, benzaldehyde, safranal, 2-pentylfuran, farnesene, and β-elemene were predominant compounds in the tested samples [72]. Lastly, in their review, Yan et al. reported that the non-polar fraction of dandelion roots contained mainly unsaturated fatty acids, and to a far smaller extent, aldehydes, alcohols, sesquiterpenes, and monoterpenes [10].

## 3. Materials and Methods

### 3.1. Plant Material and Reagents

Dried *T. officinale* flowers (DF), purchased from “DKfromlinen” (Kaunas, Lithuania), were ground using an ultra-centrifugal mill ZM 200 (Retsch, Haan, Germany) with a 0.5 mm sieve and stored at room temperature in sealed glass containers until further analysis.

Folin–Ciocalteu’s phenol reagent (2M) from Buchs, Switzerland; Trolox (6-hidroksi-2,5,7,8-tetramethylchroman-2-carboxylic acid, 97%), gallic acid (3,4,5-trihydroxybenzoic acid, 99%), and methanol (≥99.9%) were purchased from Sigma-Aldrich (Steinheim, Germany); Na_2_CO_3_ from Chempur, Poland); ABTS reagent ((2,2′-azino-bis-(3-ethylbenzothiazoline-6-sulfuronic acid) diammonium salt, ≥98%), and β-carotene (≥93%) were purchased from Sigma-Aldrich (St. Louis, MO, USA); sodium chloride (NaCl), potassium dihydrophosphate (KH_2_PO_4_), sodium hydrophosphate (Na_2_HPO_4_), potassium chloride (KCl), and ammonium acetate (CH_3_COONH_4_) from Reachem (Bratislava, Slovakia); potassium persulfate (K_2_S_2_O_8_) from Lach-Ner (Neratovice, Czech Republic); neocuproine (2,9-dimethyl-1,10- phenanthroline, ≥98%) from Sigma-Aldrich (Wuxi, China); copper chloride dihydrate (CuCl_2_∙2H_2_O) from Thermo Fisher Scientific (Kandel, Germany); aluminium chloride (AlCl_3_) from UAB “Eurochemicals” (Vilnius, Lithuania); quercetin from Cayman Chemical Company (Ann Arbor, MI, USA); ethanol (96%, food grade) from AB “Vilniaus degtinė” (Vilnius, Lithuania); carbon dioxide and nitrogen gases (99.9%) from “Gaschema” (Jonava, Lithuania). All solvents were of analytical or HPLC-grade.

### 3.2. SFE-CO_2_ Extraction of T. officinale Flowers

The SFE-CO_2_ (with and without the addition of 5% EtOH as co-solvent) of DF was performed using an SFT-110 extraction system (Supercritical Fluid Technologies, Newark, DE, USA). For the extractions, 15.000 ± 0.001 g of ground material (0.5 mm) was placed in a 50 mL cylindrical extractor (38 mm inner diameter, 136 mm length) between two layers of cotton wool to prevent particle transfer to the system. The temperature of the cylindrical extractor was regulated by a heating cover surrounding it. The extraction conditions were as follows: pressure, 35 MPa; temperature, 40 °C; dynamic extraction with continuous supercritical CO_2_ flow was performed for 195 min, with extraction yield measured every 15 min. Based on previous studies by our research group, each dynamic extraction experiment was preceded by a 10 min static extraction. All experiments were carried out manually using a ball float rotameter to maintain a CO_2_ flow rate of 1.8–2.2 SL/min (standard litres per minute at standard conditions: P_CO2_ = 100 kPa, T_CO2_ = 20 °C, ρ_CO2_ = 0.0018 g/mL) [73,74].

For yield comparison, Soxhlet extraction with hexane (SOX-He) was performed using 3.000 ± 0.001 g of DF (solid-to-liquid ratio 1:83) in an automated Soxhlet extractor EZ100H (Behr Labor-Technik, Düsseldorf, Germany) under reflux at 68 °C and atmospheric pressure, with an extraction rate of 1 cycle/5 min for a total duration of 6 h. After extraction, hexane was removed under nitrogen flow using a Büchi V–850 Rotavapor R–210 (Flawil, Switzerland), and the resulting SOX-He extract was further kept under nitrogen flow for 5 min.

Yields of SFE-CO_2_, SFE-CO_2_ + 5% EtOH, and SOX-He extracts were determined gravimetrically (±0.001 g) and expressed in g/100 g DF; extracts were placed in dark glass bottles and stored in the freezer (−20 °C) before the analysis. Extraction experiments were performed in triplicate.

### 3.3. In Vitro Antioxidant Capacity Assessment

The in vitro antioxidant capacity of DF extracts (E) was assessed using the cupric ion reducing antioxidant capacity (CUPRAC) and the ABTS^•+^ assays, as reported by our group previously [75,76]. All analyses were performed in quadruplicate, and absorbances were measured using a GENESYS 50 UV-Vis Spectrophotometer (Thermo Fisher Scientific, Waltham, MA, USA).

For the CUPRAC assay, 400 µL of DF extract (SFE-CO_2_: 0.25 mg/mL; SFE-CO_2_ + 5% EtOH: 0.125 mg/mL) or blank (EtOH) was mixed with 400 µL of CuCl_2_ (1 mM) solution, 400 µL of neocuproine (7.5 mM), and 400 µL of NH_4_Ac buffer (pH 7), then kept in the dark for 30 min and the absorbance was measured at 450 nm. The results were expressed as Trolox equivalent antioxidant capacity for CUPRAC (TEAC_CUPRAC_, mg TE/g E and DF) using a dose-response curve for Trolox (25–200 µmol/L).

For the ABTS assay, 25 µL of DF extract (SFE-CO_2_: 15 mg/mL; SFE-CO_2_ + 5% EtOH: 1.5 mg/mL), or blank (EtOH) were added to a 1500 µL ABTS^•+^ solution in phosphate buffer saline (PBS; 75 mmol/L; pH 7.4) [prepared by mixing 50 mL of ABTS reagent (2 mmol/L PBS) with 200 µL K_2_S_2_O_8_ (70 mmol/L), and after 15–16 h diluting with PBS to obtain the absorbance of AU 0.700 ± 0.010 at 734 nm], mixtures were kept in the dark for 2 h and the absorbance was measured at 734 nm. Results were expressed as mg TE/g E and DF using a dose-response curve for Trolox (0–1500 µmol/L).

### 3.4. Total Phenolic Content (TPC) Assessment

Briefly, for the TPC evaluation by Folin–Ciocalteu’s assay [75,76], 150 µL of DF extract (SFE-CO_2_: 2 mg/mL; SFE-CO_2_ + 5% EtOH: 0.5 mg/mL) or blank (EtOH) was mixed with 750 µL of Folin–Ciocalteu’s reagent (2M, 1:9, *v*/*v*) and after 3 min of reaction, 600 µL of Na_2_CO_3_ solution (75 g/L), samples were then left in the dark for 2 h, and the absorbance was measured at 760 nm. The results were expressed as mg GAE/g E and DF using a dose-response curve for gallic acid (0–80 µg/mL).

### 3.5. Total Flavonoid Content (TFC) Assessment

TFC was measured using the AlCl_3_ colourimetric method reported by Vongsak et al. [77]: 500 µL of DF extract (SFE-CO_2_: 0.25 mg/mL; SFE-CO_2_ + 5% EtOH: 0.50 mg/mL) was mixed with 500 µL of 2% AlCl_3_ solution. The blank sample was prepared by mixing 500 µL of the DF extract and 500 µL of the EtOH. The mixtures were kept at room temperature for 10 min, and the absorbance was measured at 415 nm. Results were expressed as mg of quercetin equivalents (mg QE/g E and DF) using a dose–response curve for quercetin (1–20 µg/mL). Experiments were performed in quadruplicate.

### 3.6. β-Carotene Content Assessment

Following the procedure of Biswas et al. [78], the β-carotene content in the SFE-CO_2_ (0.2 mg/mL) and SFE-CO2 + 5% EtOH (0.125 mg/mL) extracts was determined by measuring the absorbance at 450 nm. Results were expressed as mg β-carotene/g E and DF using a dose–response curve for β-carotene (0–10 µg/mL). The experiments were carried out in quadruplicate.

### 3.7. UV Absorbance Test and Sun Protection Factor (SPF) Determination

The absorbance of SFE-CO_2_ and SFE-CO_2_ + 5% EtOH extracts (0.05–1 mg/mL in EtOH) was measured between 200 and 800 nm at every 1 nm, covering the UV-A (315–400 nm) and UV-B (280–315 nm) ranges. The experiments were carried out in quadruplicate. The SPF value was calculated based on the Mansur equation using the absorbance data measured in the range of 290 to 320 nm at every 5 nm, as previously reported elsewhere [75]:

$$SPF=CF\times \sum_{290}^{320}EE\;\left(\lambda \right)\times I\;\left(\lambda \right)\times Abs\;(\lambda )$$

UV−B absorption,%=100−(100÷SPF)

where: *CF*: correction factor (10); *EE*: erythemogenic effect of radiation with wavelength *λ*; *I*: solar intensity spectrum; *Abs* (*λ*): spectrophotometric absorbance values at wavelength. The values of *EE* × *I* are constant and were previously reported by Sayre et al. [79].

### 3.8. Determination of Volatile Compound Composition by GC × GC-TOF-MS

The volatile compound composition was determined using the modified method of Nagybákay et al. [80]. For the analysis, 0.100 ± 0.001 g of *T. officinale* flowers, or SFE-CO_2_, SFE-CO_2_ + 5% EtOH extracts were placed in a 20 mL SPME vial and subjected to the solid-phase microextraction (SPME) with a DVB/CAR/PDMS fibre at the following conditions: temperature 40 °C, equilibration time 15 min, extraction time 30 min. The analysis of SPME-derived samples was conducted on a comprehensive gas chromatography time-of-flight mass spectrometry (GC × GC-TOF-MS) LECO Pegasus 4D system, consisting of an Agilent 7890A GC system, a Gerstel multipurpose sampler MPS (Gerstel GmbH, Mulheim an der Ruhr, Germany) coupled with a high-speed TOF-MS detector (LECO, St. Joseph, MI, USA). The chromatographic system consisted of a primary column, BPX-5 (30 m, 0.25 mm internal diameter, 0.25 μm film thickness) (SGE Analytical Science, Australia), linked to a secondary column, BPX-50 (2.0 m, 0.10 mm internal diameter, 0.1 μm film thickness). Working conditions were as follows: desorption time 5 min; oven temperature started at 40 °C (hold 1 min) and ramped to 300 °C at 7 °C/min rate (hold 1 min); modulator offset 33 temperature 15 °C; transfer line to MSD 250 °C; the GC injector port temperature set at 180 °C then ramped to 250 °C at 720 °C/min; carrier gas (He) 1 mL/min; splitless injection; TOF-MS acquisition rate 10 spectra/s, mass range 30–500 m/z units; detector voltage 1550 V; ion source temperature 250 °C, solvent delay 400 s. Data from the GC × GC-TOFMS system were collected by ChromaTOF software v.4.22 (LECO) after a solvent peak delay of 360 s. Experiments were performed in triplicate. Volatile compounds were identified by comparing their mass spectra with those of the Adams, NIST, MainLib, and Replib mass spectral libraries (acceptable matches were defined as having a signal-to-noise ratio greater than 50 and a similarity greater than 750). The linear retention indexes (LRI) were calculated using the retention times of the C_7_-C_30_ n-alkane series and further compared with previously published data in the literature [52,53,54,55,56,57,58,59,60,61,62,63,64,65,66,67,68,69,70].

### 3.9. Statistical Analysis

GraphPad Prism 10.6.1. software (2025) was used to calculate mean values and standard deviations, and to evaluate differences between means with significant variation (*p* < 0.05) using an unpaired *t*-test or one-way ANOVA and Tukey’s test.

## 4. Conclusions

The present study demonstrates the effectiveness of SFE-CO_2_ extraction, particularly with EtOH as a co-solvent, in isolating valuable lipophilic bioactive compounds from *T. officinale* flowers. The EtOH-modified extracts demonstrated significantly enhanced antioxidant capacity, higher yields of total phenolics, flavonoids, and β-carotene, and superior photoprotective properties, as evidenced by elevated SPF values, as compared to the neat CO_2_ extract. Additionally, volatile compound profiling revealed a rich, diverse aroma-active composition, with monoterpenoids, aldehydes, and esters contributing to the sensory and potential functional qualities of the SFE-CO_2_ extracts. Overall, the findings of this study indicate the potential of dandelion flower extracts for the development of natural antioxidant and photoprotective formulations, particularly in the cosmetic and dermatological sectors. As these findings are based on in vitro assays, future studies should focus on in vivo validation of the photoprotective and antioxidant effects to confirm efficacy and safety in real-world applications. Moreover, formulation studies assessing stability, skin penetration, and synergistic interactions with other natural compounds would be valuable for product development. Mechanistic studies exploring the molecular pathways of tyrosinase inhibition and UV protection would enhance targeted applications. Ultimately, evaluating the scalability and economic feasibility of ethanol-modified SFE-CO_2_ for industrial applications is vital for translating these findings into commercial products.

## Figures and Tables

**Table 1:** Yields, in vitro cupric ion reducing antioxidant capacity (TEAC_CUPRAC_), ABTS radical scavenging activity (TEAC_ABTS_), total phenolic content (TPC), total flavonoid content (TFC), and β-carotene content of extracts obtained from *T. officinale* flowers under different SFE-CO_2_ conditions.

Samples		SFE-CO_2_	SFE-CO_2_ + 5% EtOH
		40 °C, 35 MPa, 195 min	40 °C, 35 MPa, 195 min
In vitro antioxidant activity:			
TEAC_CUPRAC_	mg TE/g E	84.42 ± 0.50 ^a^	169.78 ± 0.99 ^b^
	mg TE/g DF	4.06 ± 0.02 ^a^	12.29 ± 0.07 ^b^
TEAC_ABTS_	mg TE/g E	17.54 ± 0.60 ^a^	193.80 ± 0.65 ^b^
	mg TE/g DF	0.84 ± 0.03 ^a^	14.03 ± 0.05 ^b^
Total phenolic and flavonoid content:			
TPC	mg GAE/g E	29.12 ± 0.52 ^a^	91.30 ± 1.07 ^b^
	mg GAE/g DF	1.40 ± 0.02 ^a^	6.61 ± 0.08 ^b^
TFC	mg QE/g E	13.11 ± 0.22 ^a^	23.91 ± 0.39 ^b^
	mg QE/g DF	0.63 ± 0.03 ^a^	1.73 ± 0.03 ^b^
Pigment content:			
β-carotene	mg/g E	28.16 ± 0.13 ^a^	44.71 ± 0.24 ^b^
	mg/g DF	1.35 ± 0.01 ^a^	3.24 ± 0.02 ^ba^

TPC: total phenolic content; GAE: gallic acid equivalents; TFC: total flavonoid content; QE: quercetin equivalents; TEAC: Trolox equivalent antioxidant capacity; CUPRAC: cupric ion reducing antioxidant capacity; ABTS: ABTS^•+^ scavenging activity; TE: Trolox equivalents; E: extract; DF: *T. officinale* flowers; SFE-CO_2_: supercritical carbon dioxide extraction. Values of mg/g DF are calculated considering SFE-CO_2_ and SFE-CO_2_ + 5% EtOH yields. Results are expressed as mean ± SD (*n* = 4). Different superscript letters in the same row indicate significantly different values (*p* < 0.05) based on a two-tailed unpaired *t*-test.

**Table 2:** Sun protection factors (SPFs) of non-polar extracts obtained from *T. officinale* flowers under different SFE-CO_2_ conditions.

Extract	Concentration (mg/mL)	Sun Protection Factor (SPF)	UV-B Absorption %
SFE-CO_2_ (35 MPa, 40 °C, 195 min)	0.05	0.83 ± 0.03 ^a^	-
	0.10	1.63 ± 0.05 ^ab^	39
	0.25	3.84 ± 0.12 ^cd^	74
	0.50	7.26 ± 0.24 ^f^	86
	1.00	13.92 ± 0.45 ^g^	93
SFE-CO_2_ + 5% EtOH (35 MPa, 40 °C, 195 min)	0.05	2.96 ± 0.10 ^c^	66
	0.10	5.62 ± 0.18 ^e^	82
	0.25	13.53 ± 0.44 ^g^	93
	0.50	26.62 ± 0.86 ^h^	96
	1.00	49.51 ± 1.61 ^i^	98

Results are expressed as mean ± SD (*n* = 4). Different superscript letters in the same column indicate significantly different values (*p* < 0.05) based on a one-way ANOVA and Tukey’s test.

## Data Availability

All related data and methods are presented in this paper. Additional inquiries should be addressed to the corresponding author.
